# The Endocannabinoid System as a Potential Mechanism through which Exercise Influences Episodic Memory Function

**DOI:** 10.3390/brainsci9050112

**Published:** 2019-05-16

**Authors:** Paul D. Loprinzi, Liye Zou, Hong Li

**Affiliations:** 1 Exercise & Memory Laboratory, Department of Health, Exercise Science and Recreation Management, The University of Mississippi, Oxford, MS 38677, USA; 2 Lifestyle (Mind-Body Movement) Research Center, College of Psychology and Sociology, Shenzhen University, Shenzhen 518060, China; liyezou123@gmail.com; 3 Shenzhen Key Laboratory of Affective and Social Cognitive Science, College of Psychology and Sociology, Shenzhen University, Shenzhen 518060, China; 4 Research Centre of Brain Function and Psychological Science, Shenzhen University, Shenzhen 518060, China; 5 Shenzhen Institute of Neuroscience, Shenzhen University, Shenzhen 518060, China

**Keywords:** BDNF, CB1, CB2, episodic memory, exercise

## Abstract

Emerging research demonstrates that exercise, including both acute and chronic exercise, may influence episodic memory function. To date, mechanistic explanations of this effect are often attributed to alterations in long-term potentiation, neurotrophic production, angiogenesis, and neurogenesis. Herein, we discuss a complementary mechanistic model, suggesting that the endocannabinoid system may, in part, influence the effects of exercise on memory function. We discuss the role of the endocannabinoid system on memory function as well as the effects of exercise on endocannabinoid alterations. This is an exciting line of inquiry that should help delineate new insights into the mechanistic role of exercise on memory function.

## 1. Introduction

**Figure 1:**
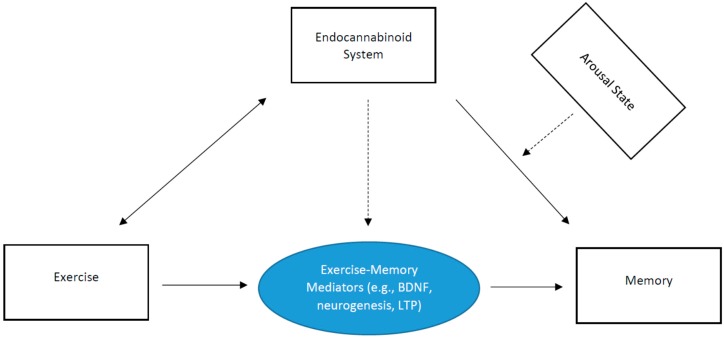
Schematic depicting the role of the endocannabinoid system on the exercise-memory interaction. The dashed lines indicate a moderation effect.

The purpose of the present review, written in a brief format, is to discuss a new potential mechanistic paradigm (endocannabinoid system) to elucidate the effects of exercise on episodic memory. This review is structured by first discussing the effects of exercise on memory; then briefly discussing the endocannabinoid system; then indicating the role of the endocannabinoid system on memory function; then how exercise may alter the function of the endocannabinoid system; and then lastly, introducing a hypothetical model indicating the potential moderational role of the endocannabinoid system on the exercise-memory interaction. This review is not meant to be an exhaustive review of the literature. Rather, the goal is to discuss a new mechanistic model and then succinctly provide support for the pathways within our model (Figure 1). Ultimately, the goal of this paper is to discuss a new mechanistic insight to help spawn the development of additional work in this important area of research.

## 2. Effects of Exercise on Memory

Emerging research from our laboratory demonstrates that exercise, including both acute and chronic exercise, may be effective behaviors in enhancing memory function [1,2,3,4,5,6,7,8,9]. Various mediators of this exercise-memory interaction have been proposed [10,11,12]. From a chronic exercise perspective, potential mechanisms may occur at multiple levels, including molecular, cellular, and structural levels. At the molecular level, and as we have thoroughly detailed elsewhere [13,14,15,16], chronic exercise may increase levels of brain-derived neurotrophic factor (BDNF) [15,16], vascular endothelial growth factor (VEGF), insulin-like growth factor-1 (IGF-1) [14], and astrocytes [13]. These molecular alterations may induce cellular changes, including gliogenesis, neurogenesis, synaptogenesis, and angiogenesis. These cellular changes, in turn, may alter structural and functional adaptations, including increased white matter, gray matter, receptor activity, neural activity, and cerebral blood flow. Collectively, these molecular, cellular and structural/functional adaptations may improve behavioral performance in memory function.

From an acute exercise perspective, which we have discussed in detail elsewhere [10,11,15,17], various exercise-induced alterations may help facilitate long-term potentiation, a cellular correlate of episodic memory [18]. Acute exercise, via, for example, muscle spindle activation, may increase neuronal excitability in key memory-related brain structures (e.g., hippocampus). This increased neuronal excitability may increase central levels of BDNF, which may help upregulate the expression and function of NMDA receptors. Downstream of this BDNF/TrkB signaling pathway, activation of the PI3K/AKT pathway may contribute to the maintenance of long-term potentiation via NMDA activity [19].

The present paper builds on our previous discussions of potential mechanisms through which exercise influences memory. That is, here we discuss a unique role of the endocannabinoid system in influencing the effects of exercise on memory function.

## 3. The Endocannabinoid System

Detailed information on the endocannabinoid system can be found elsewhere [20,21]. The cannabinoid system contains two notable subtypes of G protein-coupled receptors, namely CB1 and CB2. The role of endocannabinoids on cognitive processes has mainly focused on CB1 receptors, which are widely distributed throughout the brain and body. CB1 receptors are distributed in the CNS (brainstem, cortex, nucleus, accumbens, hypothalamus, cerebellum, hippocampus, amygdala, spinal cord) and periphery (immune system, liver, bone marrow, pancreas, lungs, vascular system, muscles, GI tract, and reproductive organs) [22]. CB2 receptors are also distributed in the CNS (brainstem, glial cells) and periphery (immune system, liver, bone marrow, pancreas, spleen, bones, skin) [22].

## 4. The Endocannabinoid System and Memory Function

Previous reviews have detailed the role of the endocannabinoid system on memory function [22,23,24]. The influence of cannabinoids in memory function can be traced back to early work showing that marijuana intoxication (delta-9-tetrahydrocannabinol, THC) disrupts short-term memory function [25]. Such effects of THC on memory impairment appear to occur in a dose-dependent manner [26,27], with this disruption occurring primarily in the dentate gyrus, where high densities of cannabinoid receptors exist [28], and exist mainly in GABA-ergic inhibitory neurons. Further, memory impairment effects from marijuana may occur, in part, from its detrimental effects on information processing and reduced blood flow to the temporal lobe [24].

Acute systemic administration of CB1 agonists has been shown to impair acquisition of memory across multiple memory tasks, including the Morris water maze task [29]. Similar results have also been observed with intra-cranial administration of CB1 agonists [30]. Conversely, administration of antagonists of CB1 receptors has been shown to facilitate memory consolidation [31]. Blockage of CB1 receptors increases the release of acetylcholine (ACh) [32], a neurotransmitter essential for memory and learning.

Cannabinoid receptor activation may impair memory through various pathways. For example, activation of CB1 receptors is connected with inhibition of adenyl cyclase as well as calcium channels and leads to the activation of potassium channels [22]. As a result, this leads to short-term depression of neurotransmitter release. More specifically, CB1 receptor activation may inhibit cAMP accumulation within neurons, inhibit glutamate release, and inhibit voltage-activated calcium currents [33,34,35], of which may reduce the excitability of hippocampal neurons, and in turn, reduce neural transmission [32]. Further, cannabinoid agonists may interfere with long-term potentiation [36]. Notably, however, previous work has shown that pre-incubation of adult rat hippocampal slices with THC can either inhibit or potentiate long-term potentiation, depending on the concentration used [37]. Possible explanations for contrasting results for THC on memory is that cannabinoid receptors are expressed at both glutamatergic and GABA-ergic synapses, which often exert opposite effects on memory [38]. For example, CB1 activation from low doses impacts glutamatergic transmission, whereas higher doses affect GABA-ergic transmission [39]. Relatedly, a chronic low dose of THC has been shown to reverse age-related decline in cognitive performance, via enhanced expression of synaptic markers and increased hippocampal spine density [40]. Further, cannabinoid-induced depression of synaptic transmission is switched to stimulation when dopaminergic tone is increased [41]. Although less investigated than CB1 receptors, recent work suggests an important role of CB2 receptors in memory function [42]. Chronic activation of CB2 receptors in the hippocampus for 7–10 days has been shown to increase excitatory synaptic transmission [43]. Similarly, other related work demonstrates that CB2 receptors play an important role in the modulation of memory consolidation for aversive experiences [44]. Further, CB2 receptor agonists reduce neurodegeneration, neuroinflammation, and attenuates spatial memory impairment in an Alzheimer’s disease model [45]. Relatedly, CB2 knockout has been shown to impair contextual long-term memory [46]. In addition to direct activation of CB2 receptors, other work also demonstrates the important role of key enzymes (e.g., fatty acid amide hydrolase, FAAH) that are responsible for the metabolism of key endocannabinoids (e.g., anandamide) [47]. For example, recent work has shown that FAAH inhibition modulates hippocampal microglial recruitment and activation that is associated with improved hippocampal-dependent memory [48]. Relatedly, FAAH inhibitor (URB597) infusion, which selectively increased anandamide levels at active synapses, enhanced emotional memory via consolidation-based processes [49]. Treatment with URB597 has also been shown to restore age-related decreases in long-term potentiation in the dentate gyrus [50].

The conflicting findings of the endocannabinoid system on memory function may also be context-dependent. As thoroughly detailed elsewhere [44], the endocannabinoid system may shape how environmental stimuli influence emotional responses. In a low arousal state, endocannabinoid activation was not associated with memory in rats, which was in contrast to their findings in a high arousal state, showing that short-term memory was enhanced when endocannabinoid activation occurred during the early memory consolidation stage [45]. Thus, environmental or behavioral events that influence different levels of stress and arousal may shape the responses to the memory effects of the cannabinoid system. As detailed elsewhere [44], emotionally arousing experiences, such as stress and physical exercise, increase stress hormones (e.g., cortisol and epinephrine), which bind to metabotropic receptors within the basolateral complex of the amygdala, activating the cAMP/PKA pathway to induce endocannabinoid synthesis. Endocannabinoids are then released, bind to GABAergic terminals, inhibits GABA release, and in turn, increase noradrenergic activation of postsynaptic β-adrenoceptors, ultimately facilitating memory consolidation of emotional/arousing events. These effects may, in part, help explain the potential beneficial effects of exercise on memory function. This may be particularly true for studies evaluating the effects of exercise on emotional memory. As we demonstrated recently [51], when exercise occurs during the memory consolidation stage, emotional memory is enhanced, whereas when it occurs prior to memory encoding, it remains unaffected [52].

## 5. Exercise and the Endocannabinoid System 

Several studies have demonstrated that endocannabinoid levels may be altered with exercise [53,54], with their effects acting both centrally and peripherally [54]. Exercise has been shown to enhance CB1 receptor sensitivity [55]. Sparling et al. [56] demonstrated that higher levels of physical activity were associated with greater anandamide (an endogenous agonist of the cannabinoid CB1 and CB2 receptors) levels. Among rodents, Hill et al. [57] showed that 8 days of exercise increased anandamide levels. Further, Raichlen et al. [58] showed an intensity-dependent effect of exercise on anandamide levels, with moderate-intensity exercise enhancing anandamide levels. Fuss et al. [59] showed that wheel running increases endocannabinoid levels and ablation of CB1 receptors on GABAergic neurons inhibits running-induced anxiolysis. Notably, however, a bi-directional relationship may also exist, as research demonstrates that stimulation of CB1 receptors is a prerequisite for voluntary running in mice [60,61,62]. For example, CB1 activation on VTA (ventral tegmental area) GABAergic neurons may trigger disinhibition of VTA dopamine [60], implicated in reward-directed processes.

## 6. Hypothetical Model

Emerging work has started to evaluate the potential role of the cannabinoid system on subserving the exercise-memory relationship. Research demonstrates that exercise-induced hippocampal cell proliferation and neurogenesis depends on CB1 receptor signaling [57,63,64]. Notably, CB1 receptors have widespread expression over the entire dentate gyrus and voluntary wheel running has been shown to increase CB1 receptor mRNA in the hippocampus [63]. CB1 receptors specifically affect the stages of adult neurogenesis and the survival and maturation of new neurons [63].

Relatedly, research demonstrates that treadmill running improves spatial memory in mice, which is prevented by simultaneous treatment of a CB1 receptor antagonist [65]. Such exercise-related effects may be attributed to exercise-induced increases in CB1 receptor activation and BDNF expression in the hippocampus [65]. Thus, exercise-induced enhancement of memory function may, in part, be due to a number of endocannabinoid signaling mechanisms related to long-term potentiation, production of neurotrophic factors, and cellular neurogenesis. This is schematically illustrated in Figure 1. That is, there exists a bi-directional relationship between exercise and endocannabinoid levels. The endocannabinoid system may play an important role in episodic memory function, and as demonstrated previously, this may be moderated by arousal state. Further, key exercise-induced mechanisms (e.g., neurogenesis) that influence episodic memory function may be moderated by the endocannabinoid system. Key insight and support of this model have been demonstrated recently. Bosch et al. [66] evaluated the effects of acute exercise intensity on memory function, with considerations of AEA (anandamide) and BDNF in mediating this relationship. Their results demonstrated consistent evidence of moderate-intensity acute exercise enhancing associative memory. They also demonstrated that increased AEA after moderate-intensity exercise correlated with neural activation of the right hippocampus [66].

## 7. Model Evaluation

Future work is needed to evaluate this model and, when appropriate, make necessary revisions. Such work should employ both acute and chronic exercise paradigms. From an acute exercise perspective, future within-subject experimental designs should employ multiple exercise intensities (e.g., control, moderate, and vigorous), and when doing so, carefully consider the temporal effects of acute exercise on memory function [4]. That is, consider integrating the acute bout of exercise prior to memory encoding and across different phases of memory consolidation. In human models, blood samples to assess endocannabinoid levels should be measured at multiple time points (e.g., before and after exercise; prior to memory encoding and retrieval, and during memory consolidation). Similarly, key mediators (e.g., BDNF, LTP) through which the endocannabinoid system may influence the effects of acute exercise on memory will need to be assessed at these time points. In human work, novel methodologies to assess LTP will need to be considered. For example, evaluating LTP-like responses, such as visually-evoked event-related potentials, is worth considering [67]. Further, the memory assessments should be carefully considered, and, for example, include hippocampal-dependent memory tasks and emotional memory tasks (given the abundance of CB1 receptors in the limbic system).

Among human models, chronic exercise training studies should carefully design the study to ensure that any potential effects are due to the chronic training stimulus, as opposed to a potential acute exercise response. Rarely do chronic training studies indicate whether participants avoided exercise shortly before the post-training memory assessment, and as such, it is challenging to determine whether post-training outcomes are from chronic adaptations from exercise, or rather, are an artifact of an acute exercise response. These chronic training studies should evaluate other potential mediators through which the endocannabinoid system may influence, such as neurogenesis, which can be measured from magnetic resonance imaging [68]. Lastly, animal studies should continue to design experimental studies that evaluate whether exercise activates the endocannabinoid system, whether this activation is associated with memory function, and whether blocking the endocannabinoid system prevents a direct effect of exercise on memory function.

## 8. Summary

In conclusion, this brief narrative review highlights the potential role of the cannabinoid system on the exercise-memory relationship. Future research is needed to fully test out this potential mechanistic paradigm. Such work should also delineate whether the site of CB1 activation (e.g., GABA-ergic, glutamatergic) moderates this relationship. This is an exciting line of inquiry that should help delineate new insights into the mechanistic role of exercise on memory function.
